# Pathogen Detection in Early Phases of Experimental Bovine Tuberculosis

**DOI:** 10.3390/vetsci11080357

**Published:** 2024-08-07

**Authors:** Mitchell V. Palmer, Carly Kanipe, Soyoun Hwang, Tyler C. Thacker, Kimberly A. Lehman, Nicholas A. Ledesma, Kristophor K. Gustafson, Paola M. Boggiatto

**Affiliations:** 1 Bacterial Diseases of Livestock Research Unit, National Animal Disease Center, United States Department of Agriculture, 1920 Dayton Ave, Ames, IA 50010, USA; carly.kanipe@usda.gov (C.K.); paola.boggiatto@ars.usda.gov (P.M.B.); 2 Center for Veterinary Biologics, Animal and Plant Health Inspection Service, United States Department of Agriculture, Ames, IA 50010, USA; soyoun.hwang@usda.gov; 3 National Veterinary Services Laboratories, Animal and Plant Health Inspection Service, United States Department of Agriculture, Ames, IA 50010, USA; tyler.thacker@usda.gov (T.C.T.); kimberly.lehman@usda.gov (K.A.L.); nicholas.a.ledesma@usda.gov (N.A.L.); kristophor.k.gustafson@usda.gov (K.K.G.)

**Keywords:** bovine, diagnosis, *Mycobacterium bovis*, pathogen, tuberculosis

## Abstract

**Simple Summary:**

Bovine tuberculosis is caused by *Mycobacterium bovis*, which causes tuberculosis in humans and animals. Diagnosis of bovine tuberculosis has relied on examination of immune responses to *M. bovis*; however, using these methods, disease detection during the earliest phases of infection has been difficult, allowing a window for cattle-to-cattle transmission to occur within a herd. Alternative means of diagnosis could include methods to detect *M. bovis* or *M. bovis* DNA in bodily fluids such as nasal secretions, saliva, or blood rather than the animal’s response to infection. DNA-from *M. bovis* was detected in nasal swabs and saliva from a small number of experimentally infected calves during the first 8 weeks after experimental infection. Although DNA from *M. bovis* could be detected, no culturable *M. bovis* was recovered from nasal swabs or saliva. Moreover, *M. bovis* DNA was not found in blood samples collected weekly. Successful infection of all calves was demonstrated using an interferon gamma release assay. Identification of infected animals through the detection of *M. bovis* will require the use of alternative samples or alternative assays.

**Abstract:**

Bovine tuberculosis is caused by *Mycobacterium bovis*, a member of the *M. tuberculosis* complex of mycobacterial species that cause tuberculosis in humans and animals. Diagnosis of bovine tuberculosis has relied on examinations of cell-mediated immune responses to *M. bovis* proteins using tuberculin skin testing and/or interferon gamma release assays. Even when using these methods, disease detection during the earliest phases of infection has been difficult, allowing a window for cattle-to-cattle transmission to occur within a herd. Alternative means of diagnosis could include methods to detect *M. bovis* or *M. bovis* DNA in bodily fluids such as nasal secretions, saliva, or blood. During the first 8 weeks after experimental aerosol infection of 18 calves, *M. bovis* DNA was detected in nasal swabs from a small number of calves 5, 6, and 8 weeks after infection and in samples of saliva at 1, 7, and 8 weeks after infection. However, at no time could culturable *M. bovis* be recovered from nasal swabs or saliva. *M. bovis* DNA was not found in blood samples collected weekly and examined by real-time PCR. Interferon gamma release assays demonstrated successful infection of all calves, while examination of humoral responses using a commercial ELISA identified a low number of infected animals at weeks 4–8 after infection. Examination of disease severity through gross lesion scoring did not correlate with shedding in nasal secretions or saliva, and calves with positive antibody ELISA results did not have more severe disease than other calves.

## 1. Introduction

Bacteria of the genus *Mycobacterium* are Gram-positive, acid-fast bacilli (AFB). *Mycobacterium tuberculosis* and *Mycobacterium bovis* are both members of the *M. tuberculosis* complex of mycobacterial species that can cause tuberculosis in humans and animals [1]. Tuberculosis (TB) in humans is generally caused by *M. tuberculosis*; however, the zoonotic pathogen, *M. bovis*, can produce tuberculosis in humans indistinguishable from that caused by *M. tuberculosis* [2]. Of the numerous mycobacterial species, the host range of *M. bovis* is broadest and includes many mammalian species, most notably cattle.

At the beginning of the 20th century, TB was the leading cause of death in the United States (US) [3]. Although the precise proportion is not known, it is estimated that 10–30% of human TB cases in the US and Europe were the result of contact with cattle or cattle products [3,4]. Prompted by both animal and human health concerns, the US Department of Agriculture (USDA) initiated a bovine TB (bTB) eradication program in 1917, which is still in place today. A cornerstone of that program has been in vivo animal testing, chiefly the tuberculin skin test (TST) and more recently interferon gamma release assays (IGRAs). While both methodologies measure the cell-mediated responses of the host to *M. bovis*, both fail to identify cattle during the earliest stages of infection.

Alternative diagnostic platforms, which identify the pathogen rather than the host response to the pathogen have been proposed [5]. The protracted length of the disease and the lack of clinical signs following infection allow time for the pathogen to spread within a herd without detection. The transmission of *M. bovis* among cattle may occur through either direct or indirect contact [6]. Therefore, early identification of infected cattle would be ideal to reduce cattle-to-cattle transmission. The detection of infected cattle through the examination of nasal and oral swabs has yielded variable results [7,8,9,10,11]. This is likely due to the unknown exposure dose or duration of infection in naturally infected animals and the variable dosages, routes, and sampling times used in experimental infections. Experimental infection studies have used the intranasal and intrabronchial routes of infection; however, fewer have used the aerosol route of inoculation, arguably a route that more closely mimics natural exposure. The objective of this study was to examine various bodily fluids for the presence of *M. bovis* during the first 8 weeks after experimental aerosol infection to determine if these could serve as effective diagnostic samples for the detection of infected animals. We hypothesize that in cattle infected via aerosol, mimicking natural infection, *M. bovis* will be detectable in one or more bodily fluids and could act as a means of early diagnosis of bovine tuberculosis.

## 2. Materials and Methods

### 2.1. Animals, Inoculum, and Mycobacterium bovis Aerosol Challenge

Twenty-four Holstein steers (6 months of age) were obtained from a source with no history of *M. bovis* infection. The experiment was conducted in triplicate, each replicate being composed of six *M. bovis*-infected and two control non-infected steers. Steers from replicates 1, 2, and 3 were infected with 9.7 × 10^3^ CFU/mL, 1.82 × 10^4^ CFU/mL, and 8.5 × 10^3^ CFU/mL, respectively. Aerosol infection of steers with virulent *M. bovis* has been described in detail previously [12,13,14]. In brief, the nebulization apparatus consisted of a compressed air tank and a commercially available aerosol delivery system (Equine AeroMask^®^, Trudell Medical International, London, ON, Canada) comprised of a jet nebulizer (Whisper Jet, Marquest Medical Products, Englewood, CO, USA), holding chamber, and mask. Upon inspiration, the nebulized inoculum was inhaled through a one-way valve into the mask and directly into the nostrils. A rubber gasket sealed the mask securely to the muzzle preventing leakage of inoculum around the mask. Expired air exited through one-way valves on the sides of the mask. The nebulization process continued until all the inoculum, a 1 mL phosphate-buffered saline (PBS) wash of the inoculum tube, and an additional 2 mL PBS were delivered (approximately 12 min).

This experiment used *Mycobacterium bovis* strain 10-7428, a field strain of low passage (≤3) and known virulence [13]. Inoculum was prepared as described [15] in Middlebrook’s 7H9 liquid media (Becton, Dickinson and Company, Franklin Lakes, NJ, USA) supplemented with 10% oleic –albumin–dextrose catalase (OADC; Difco, Detroit, MI, USA) plus 0.05% Tween 80 (Sigma Chemical Co., St. Louis, MO, USA). At the point of mid log-phase growth, the bacilli were pelleted by centrifugation at 750× *g*, washed twice with PBS (0.01 M, pH 7.2), and stored at −80 °C until used. Upon use, frozen stock was warmed to room temperature (RT) and diluted to the appropriate cell density in 2 mL of PBS. Bacilli were enumerated by serial dilution plate counting on Middlebrook’s 7H11 selective media (Becton, Dickinson and Company).

All experimental procedures using animals were performed in compliance with recommendations in the *Care and Use of Laboratory Animals of the National Institutes of Health* and the *Guide for the Care and Use of Agricultural Animals in Research and Teaching* [16,17]. Animal-related procedures were also approved by the National Animal Disease Center Institutional Animal Care and Use Committee (IACUC) and all procedures were conducted in accordance with the approved protocol (ARS-2019-802).

### 2.2. Nasal Swabs and Saliva Collection

To evaluate shedding in respiratory secretions and saliva, nasal swabs from both nostrils were collected 7 days prior to infection and weekly thereafter for 8 weeks using Isohelix swab packs (Cell Projects Ltd., Business Park, Maidstone, UK). Saliva was collected 7 days prior to infection and weekly thereafter for 8 weeks using saliva collection devices (SuperSal, Oasis Diagnostics Co., Vancouver, WA, USA). Mycobacterial isolation and PCR detection of *M. tuberculosis* complex DNA were performed as previously described [18,19,20,21].

### 2.3. Blood Collection and PCR

Blood was collected 7 days prior to infection and weekly thereafter for 8 weeks. Blood was collected into EDTA vacutainer tubes (Becton, Dickinson and Company), transferred to 15 mL conical tubes, combined with PBS to achieve a total volume of 14 mL, and centrifuged at 1200× *g* for 30 min at RT. The buffy coat was placed on Histopaque 1077 media (Millipore Sigma, Rockville, MD, USA) and centrifuged at 1200× *g* for 30 min at RT. The mononuclear cell layer was then removed, resuspended in PBS, and centrifuged at 300× *g* for 5–10 min at RT. The supernatant was removed, and the pellet was snap frozen in liquid nitrogen and stored at −80 °C.

DNA was isolated from the mononuclear cell pellet using QIAamp Pathogen Lysis Tubes and a QIAamp UCP Pathogen Mini Kit (Qiagen, Germantown, MD, USA), according to the manufacturer’s recommendations. Real-time PCR was performed using primers and probes that targeted IS*6110* (IS6110_T), as described previously [22]. The detection of mycobacterial DNA by real-time PCR was performed using the IS6110_T primers and a 5′ Hex labeled probe (5′-**AGCCACACTTTGCGGGCACC**-3′) with a 3′ Iowa Black FQ quencher (Integrated DNA Technologies, Coralville, IA, USA). A Taqman Fast Advanced Mastermix (Thermo-Fisher, Waltham, MA, USA) was used according to the manufacturer’s directions. The real-time PCR was run in an ABI7500 (Thermo-Fisher). The detection of β-actin was used as a positive control.

### 2.4. Interferon Gamma Release Assay

Seven days prior to infection and weekly thereafter for 8 weeks, blood was collected for the evaluation of cell-mediated immune responses using commercially available assays for antigen-specific interferon gamma (IFN-γ) production. Whole blood samples were collected in sodium-heparinized tubes (Becton, Dickinson and Company) from the jugular vein and transferred to the laboratory at RT within 2 h of collection. In the laboratory, blood samples were stimulated within 10 h after collection. Samples were divided into four aliquots of 1.0 mL each in 48-well cell tissue culture plates (Thermo-Fisher). Purified protein derivative (PPD; ID Vet, Grabels, France) from *M. bovis* (PPD-B), purified protein derivative from *M. avium* (PPD-A), pokeweed mitogen (PWM; Thermo Fisher), and PBS as a nil antigen control were used for the stimulation of whole blood samples. One hundred microliters of PPD-B (0.3 μg/mL), PPD-A (0.3 μg/mL), PWM (positive control), or PBS (nil antigen) were added and mixed in each well and incubated at 37 °C with 5% CO_2_ for 16–24 h. After incubation, 48-well tissue culture plates were centrifuged for 20 min at 900× *g* at 23 °C, and the upper layer of plasma was harvested. The samples were tested in duplicate using a sandwich enzyme immunoassay (Bovigam; Thermo-Fisher) as recommended by the manufacturer. The optical density (OD) of each well was measured at 450 nm with a 620–650 nm reference filter. The mean OD of each sample was calculated and used to define the cut-off values. The OD of a sample stimulated with PPD-B minus the OD of a sample stimulated with PPD-A (OD_PPD−B_–OD_PPD−A_) (ΔOD) was used as cut-off criteria. When (OD_PPD−B_–OD_PPD−A_) was ≥0.1 the sample was considered positive.

### 2.5. IDEXX M. bovis ELISA

Seven days prior to infection and weekly thereafter for 8 weeks, blood was collected for the evaluation of humoral immune responses using a commercially available assay. The test was performed as per the manufacturer’s instructions (IDEXX Laboratories Inc., Westbrook, ME, USA). The samples, as well as kit positive and negative controls, were diluted 1:50 in Sample Diluent. The samples and controls were transferred to antigen-coated plates in duplicate. Plates were covered and incubated at RT for 1 h. After incubation, the plates were washed 3–5 times using the kit Wash Solution. Kit conjugate was then added, the plates were covered, and they were incubated for 30 min at RT. The plates were again washed and 3,3′,5,5′-tetramethylbenzidine (TMB) substrate added. The plates were covered and incubated for 15 min at RT, after which Stop Solution was added and the plates were read at 450 nm using an 800/TS microplate reader (Agilent BioTek, Santa Clara, CA, USA). The data are presented as an S/P ratio where the (OD_sample_−OD_negative control_) is divided by the (OD_positive control_−OD_negative control_). A sample was considered positive if the S/P ratio was ≥0.30.

### 2.6. Necropsy and Lesion Scoring

Calves were humanely euthanized by intravenous administration of sodium pentobarbital. Replicate 1 was euthanized between 268 and 281 days after infection, replicate 2 was euthanized between 329 and 336 days after infection, while replicate 3 was euthanized between 250 and 252 days after infection. At necropsy, all tissues were examined for gross lesions and processed for microscopic analysis, as described previously [13]. The medial retropharyngeal, mediastinal, and tracheobronchial lymph nodes were removed and closely examined for lesions by incising at 2–3 mm intervals. The caudal mediastinal lymph node was weighed, a section was taken for histopathology, and then it was reweighed and collected for quantitative culture. The individual lung lobes (accessory, right cranial, right middle, right caudal, left cranial, and left caudal) were scored on lesion severity. Each lung lobe was examined separately and sectioned at 0.5–1.0 cm intervals to detect deep parenchymal lesions. Severity was scored using a scale of 0–5. Lungs lacking any pathology were scored as a 0. Calves with less than or equal to five lesions, all with a diameter less than 10 mm were given a score of 1. Animals with 6–10 lesions and rare (<2) lesions with a diameter between 10 and 20 mm were assigned a score of 2. Calves with 11–20 lesions and occasional (3–5) lesions with a diameter between 10 and 20 mm were assigned a score of 3. If there were greater than 20 lesions or frequent (>5) lesions with a diameter between 10 and 20 mm, the lungs were scored as a 4. A score of 5 was assigned to lungs possessing one or more of the following: countless and coalescing lesions, greater than 50% of the lesions sized >10 mm in diameter, or any lesion >20 mm in diameter. Scores from each lobe were totaled to determine a total lung score for each animal.

For quantitative culture of the caudal mediastinal lymph node, the tissues were re-weighed, homogenized in phenol red broth, and decontaminated with 0.5N NaOH. After 10 min, 12N HCl was added until the phenol red indicator turned yellow, after which 1N NaOH was added by drop until the phenol indicator turned pink. The tubes were centrifuged at 750× *g* and the supernatant was discarded. Samples of the homogenized tissue were streaked in duplicate on Middlebrook 7H10 and 7H11 plates in 10-fold dilutions. The plates were incubated at 37 °C with 5% CO_2_ and examined at 30 and 60 days for growth.

### 2.7. Statistical Analysis

All responses, lesion scores, and quantitative culture results were evaluated using the nonparametric Kruskal–Wallis ANOVA with Dunn’s multiple comparison tests (GraphPad Prism 8.0, GraphPad Software, San Diego, CA, USA). The data are reported as mean ± SEM, except for quantitative culture results, which are presented as mean ± SD to better illustrate variability. For all analyses, a *p*-value < 0.05 was considered significant.

## 3. Results

### 3.1. IGRA Results

**Figure 1:**
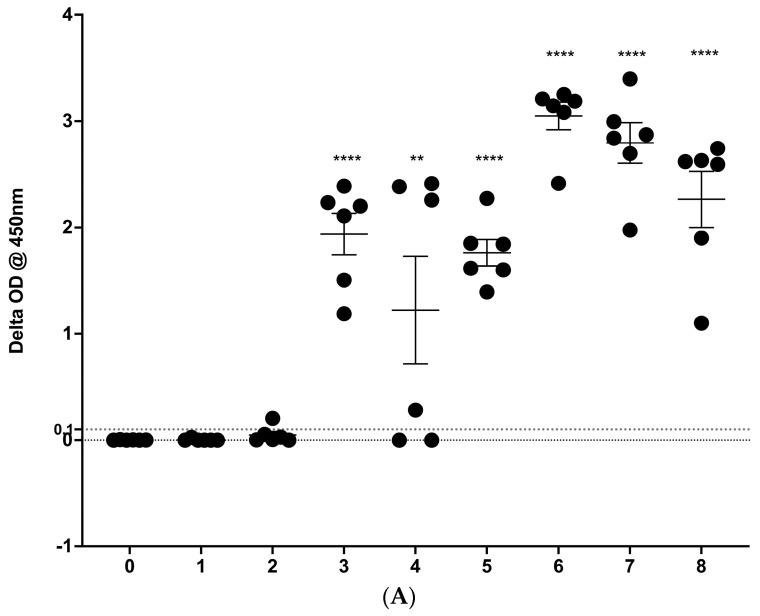

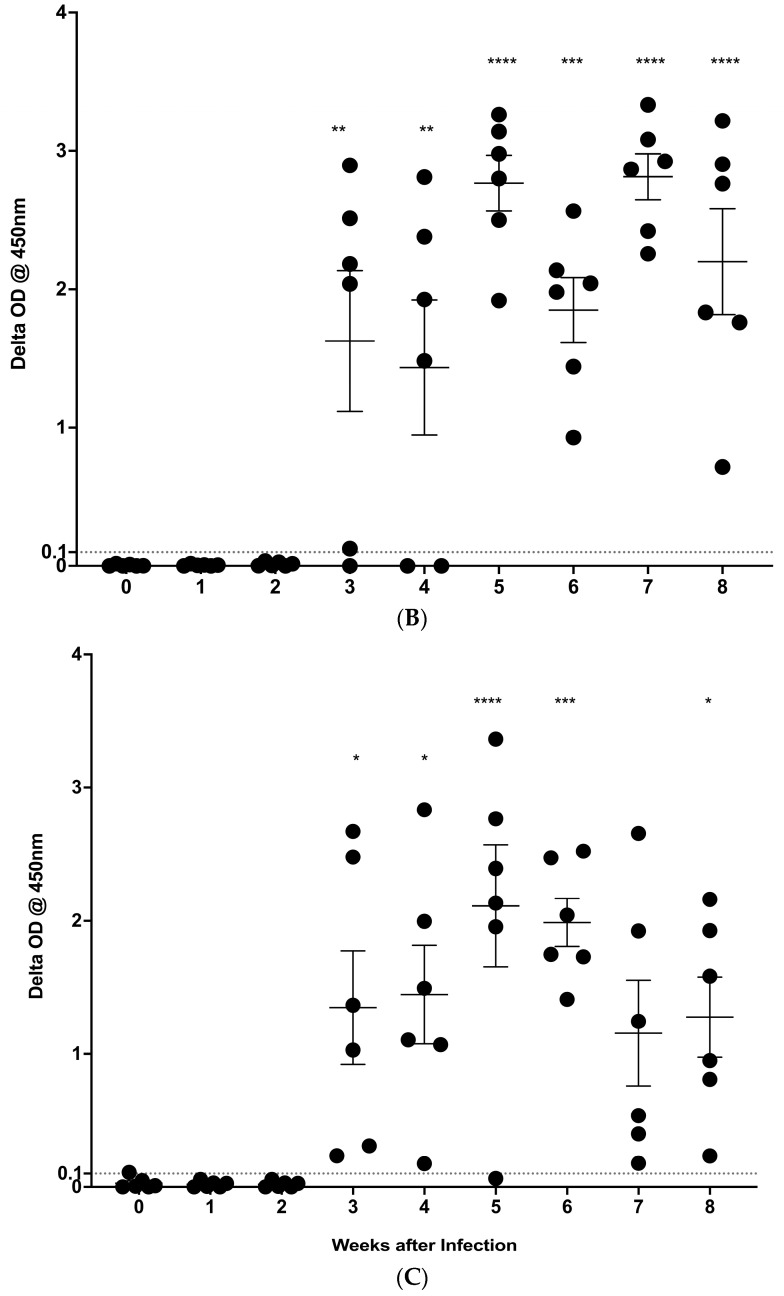
Interferon gamma release assay (IGRA) results from 3 replicates (**A**–**C**) of 6 cattle each experimentally infected with aerosolized *M. bovis* and sampled prior to infection (week 0) and weekly thereafter for 8 weeks. Results are presented as mean (PPD-B−PPD-A; ΔOD) @ 450 nm ± SEM. The cut-off for positive results is ΔOD ≥ 0.1 (dotted line). * = *p* ≤ 0.05, ** = *p* ≤ 0.001, *** = *p* ≤ 0001, and **** = *p* ≤ 00001.

During the study, all experimentally infected cattle developed IGRA responses to *M. bovis* PPD, consistent with *M. bovis* infection. The responses were similar in all three groups of cattle (Figure 1A–C). Positive IGRA responses were noted in one animal as early as week 2 in replicate 1 (Figure 1A), but by 5 weeks post-infection, all animals had positive responses. Similarly, in replicate 2, most animals developed positive IGRA responses by week 4, with all animals showing positive responses by week 5 post-infection (Figure 1B). In replicate 3, all animals had positive responses by week 3 (Figure 1C).

In all three replicates, the responses were considered statistically greater (*p* < 0.05 to <0.0001 value) than week 0 pre-infection values from week 3 through week 8, with one exception—week 7 in replicate 3 (*p* = 0.06). In individual animals, once the ΔOD values were considered positive, they remained positive, except for two animals in replicate 1 and two animals in replicate 2, which each fell below the cut-off value at week 4, and a separate single animal in replicate 3, which fell below the cut-off at week 5. All five animals returned to levels considered positive after this single time point. Based on IGRA responses, one non-infected control animal demonstrated positive results at 0 and 4 weeks. A separate animal demonstrated a positive response at week 8 (Figure S1). All other control animals remained negative throughout the study.

### 3.2. IDEXX M. bovis ELISA

**Figure 2:**
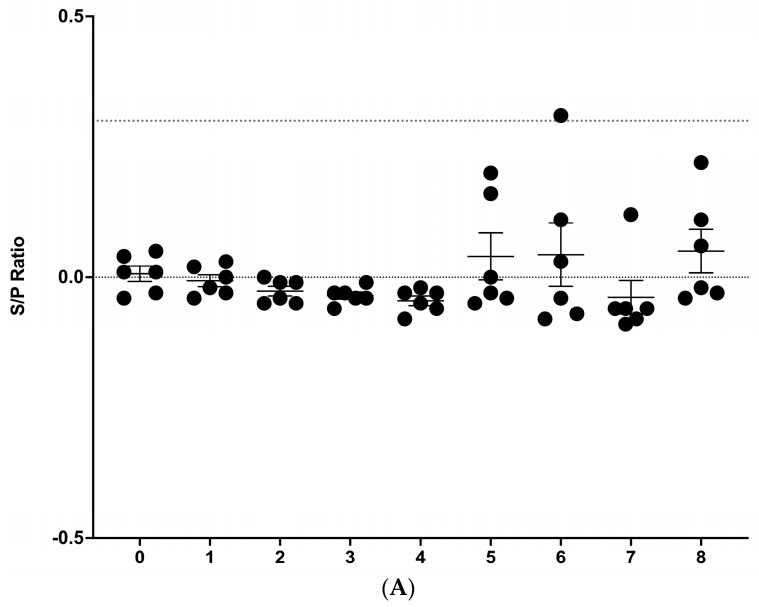

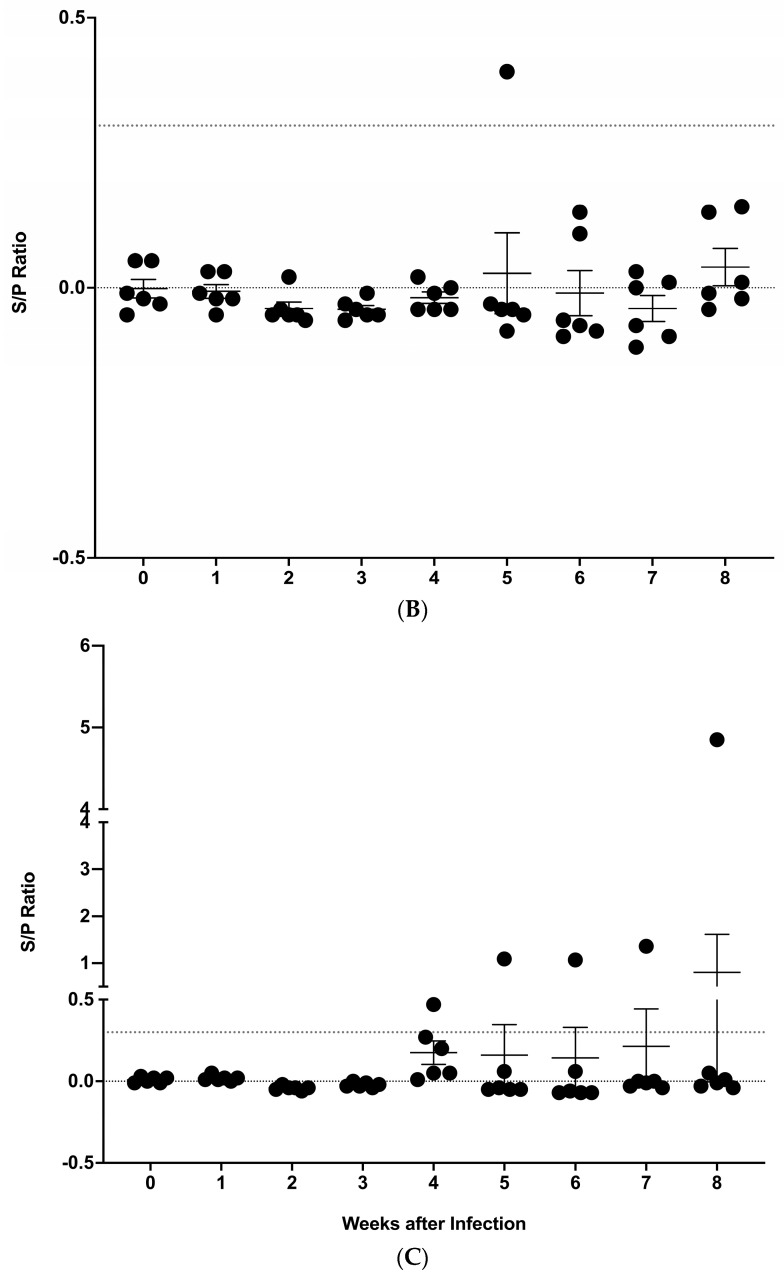
IDEXX ELISA results from 3 replicates (**A**–**C**) of 6 cattle each experimentally infected with aerosolized *M. bovis* and sampled prior to infection (week 0) and weekly thereafter for 8 weeks. The results are presented as an S/P ratio ± SEM. A sample was considered positive if the S/P ratio was ≥0.30 (dotted line).

Serological responses to *M. bovis* were also measured via the IDEXX *M. bovis* antibody kit. In all three replicates, one animal showed positive responses (S/P ≥ 0.30) in at least one time point, albeit with different degrees of magnitude (Figure 2). In replicates 1 and 2, one animal from each group showed positive responses at weeks 6 and 5 post-infection, respectively, (Figure 2A,B). In replicate 3, one animal showed positive responses starting at week 4 post-infection and remained positive at all time points analyzed, reaching its maximum response at 8 weeks post-infection with an S/P value of 5.0. This was the only animal considered positive at more than a single time point. All non-infected control animals remained negative throughout the study (Figure S2).

### 3.3. PCR Results from Nasal Swabs, Saliva and Blood

Nasal swabs collected from all animals in all three replicates showed PCR-positive results in at least one infected animal at 5-, 6-, and 8-weeks post-infection (Table 1).

These data indicate a percent detection rate of 5.55% at each time point analyzed. Similarly, in saliva samples, PCR positive results were seen from at least one infected animal at weeks 1-, 5-, 7-, and 8- post-infection (Table 2), again indicating only a 5.55% rate of detection.

Only a single animal (#68917) had both PCR-positive nasal swabs and saliva samples, which were detected at 8 weeks post-infection. At no time point during the study was culturable *M. bovis* isolated from a nasal swab or saliva sample. Similarly, at no time point was *M. bovis* DNA detected in samples of blood via real-time PCR.

### 3.4. Lesion Scoring and Quantitative Culture

**Figure 3:**
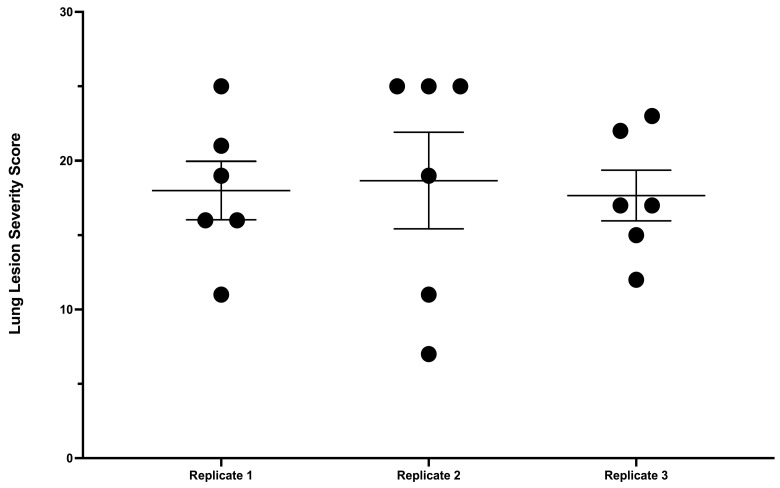
Lung lesion severity scores from steers experimentally inoculated with virulent *M. bovis* via aerosol at dosages of 9.7 × 10^3^ CFU (Replicate 1), 1.82 × 10^4^ CFU (Replicate 2), and 8.5 × 10^3^ CFU (Replicate 3). Details of scoring system are found in text.

Samples collected for microscopic analysis confirmed that gross lesions were microscopically consistent with tuberculoid granulomas of bTB. Lung lesion severity scores were similar and did not differ significantly between replicates (Figure 3). 

**Figure 4:**
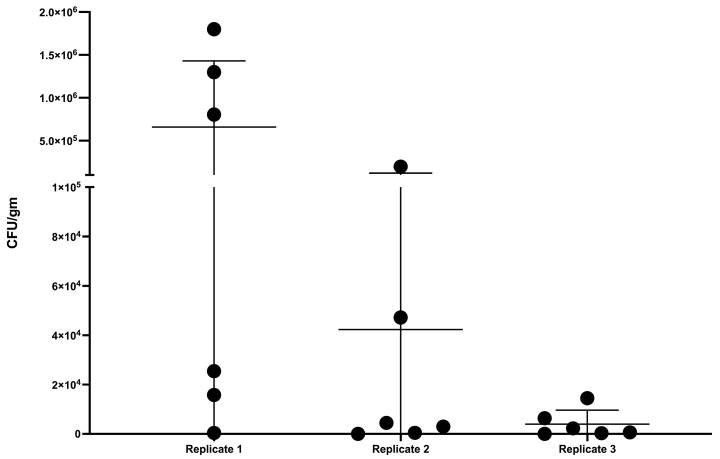
Bacterial burden (CFU/gm) ± SD of *M. bovis* in caudal mediastinal lymph nodes of steers experimentally inoculated with virulent *M. bovis* via aerosol at dosages of 9.7 × 10^3^ CFU (Replicate 1), 1.82 × 10^4^ CFU (Replicate 2), and 8.5 × 10^3^ CFU (Replicate 3). Replicate 1 was euthanized and examined 268 to 281 days after infection. Replicate 2 was euthanized and examined 329 to 336 days after infection, while Replicate 3 was euthanized and examined 250 to 252 days after infection.

There were no statistical differences between replicates in the quantitative culture results from the caudal mediastinal lymph node (Figure 4), although there was greater variability observed in replicates 1 and 2 than that of replicate 3.

There was no significant difference in lymph node weights between replicates or between replicates and controls (Figure S3), although weights were generally higher in infected animals. There was a modest negative correlation (−0.83; *p* = 0.058) between CFU/gm and mediastinal lymph node weight in replicate 1 (Figure S4). There were no significant differences in lesion scores, CFU/gm in caudal mediastinal lymph nodes, or caudal mediastinal lymph node weights between cattle that had positive PCR results on nasal swabs or saliva samples compared to those that did not.

## 4. Discussion

The results of the present study suggest that in the first 8 weeks after experimental aerosol exposure to virulent *M. bovis*, bacterial shedding in nasal secretions or saliva is infrequent, intermittent, and occurs at very low levels, contrary to our hypothesis. This is evidenced by a few PCR positive samples, all of which had relatively high Ct values (range 34.67–37.49), combined with a lack of bacteriological isolation of *M. bovis* from these samples.

Previous studies under field conditions have reported that shedding of *M. bovis* in naturally infected cattle is infrequent. The detection of *M. bovis* in nasal or oral secretions was reported in 6% of 40,000 tuberculin reactors in the Netherlands, 15.6% in one herd in India, and 9.3% in a herd in Argentina (reviewed in [11]). In one Northern Ireland abattoir, following the examination of 55 tuberculin reactors, *M. bovis* was isolated from samples of nasal mucous and/or tracheal mucus from seven cattle, originating from six different farms indicating a 12.7% detection rate [23]. Similarly, in another abattoir survey, four of twenty-five (16%) confirmed tuberculous cattle were shedding *M. bovis* in nasal secretions, detected by culturable *M. bovis* on nasal swabs [24]. Altogether, these data would suggest that detection of *M. bovis* from nasal or oral secretions may not be an effective tool for the diagnosis of infection.

One disadvantage to studying naturally infected animals is that the timeline of infection is unknown. The duration of infection may have a significant impact on bacterial shedding. This may explain, in part, the lack of consistent detection of *M. bovis* shedding under field conditions. However, experimental studies have also demonstrated a lack of consistency.

In one study by Cassidy et al., *M. bovis* was recovered from nasal mucus samples 7–14 days after intranasal infection and from tissues of the nasal mucosa (i.e., nasal turbinates, nasal septum, and pharynx) as early as 7 days and up to 42 days after infection. The animals in this study were inoculated intranasally with 1 × 10^7^ CFU of *M. bovis* given on two consecutive days [7]. On the day of the second inoculation, uninfected calves were introduced and co-housed in close contact with the intranasally infected calves. Samples of nasal mucus from the introduced calves yielded culturable *M. bovis* by day 7 of contact [8]. Similarly, in another contact study, nasal swabs from non-infected cattle housed with intranasally infected cattle yielded culturable *M. bovis* after approximately 80–90 days of contact [11]. Altogether these data suggest that at higher doses of infection, 10^4^ to 10^7^ CFU of *M. bovis*, administered intranasally, shedding can be detected directly or indirectly using contact cattle.

In other studies, using intranasally inoculated calves receiving 10^4^ or 10^6^ CFU, shedding was documented after a lag period of 7–30 days, depending on dose [9,10,11]. Of note, shedding was never documented in calves intranasally inoculated with a low dose of 92 CFU [10]. In a recent study using an endobronchial route of infection and a dose of 5 × 10^3^ CFU of *M. bovis*, no *M. bovis* DNA nor culturable *M. bovis* was seen in nasal swabs during a 10-week period after infection; however, a fecal sample yielded culturable *M. bovis* from one animal, 5 weeks after infection [25]. In separate studies using the calf aerosol model, as described here, nasal infection has rarely been reported, and then only when higher doses (10^5^ CFU) have been used [14].

The route of administration may be an important variable in the detection of *M. bovis* shedding. Thus far, in experimental infection studies, the earliest detection of *M. bovis* in nasal secretions has been seen in cattle inoculated intranasally or naïve cattle housed with intranasally inoculated cattle. Studies using intrabronchial or aerosol inoculation methods have resulted in minimal shedding of *M. bovis* and at later time points than those seen in intranasally inoculated cattle. Using the intrabronchial challenge model, inoculum is placed directly in main stem bronchi, physically bypassing the upper respiratory tract [26]. In the aerosol challenge model, tiny droplet nuclei (<5 mm) pass by the upper respiratory tract to be deposited deep in terminal bronchioles [14]. Bypassing the upper respiratory tract in the intrabronchial and aerosol challenge models likely reduces the possible colonization of the nasal and pharyngeal regions and the ability to detect *M. bovis* on nasal swabs.

Other variables that may affect the kinetics of *M. bovis* shedding in tuberculous cattle include the animal’s age, genetics, parturition, or lactation status. Surveys in bTB endemic regions demonstrated that lactating pregnant dairy cattle were more likely to be TST-positive than younger cattle or non-pregnant lactating cows [27]. Additionally, separate surveys found that both pregnant and non-pregnant lactating cows were more likely to be TST positive than non-lactating cows and that older cows were more likely to be TST-positive than younger stock [28,29]. Lactation in cattle, with its associated high metabolic demand increases disease susceptibility to infection. As early as 1951, Theobald Smith recognized that parturition in cattle was frequently followed by the generalization of local tuberculosis, rapid decline, and death [30]. Later, scientists examining specific immune responses demonstrated the immunocompromised state of periparturient cows and increased susceptibility to infections such as mastitis [31,32]. Griffin noted in pregnant red deer (*Cervus elaphus*) a significantly higher incidence of TB in pregnant hinds and attributed it to decreased cell-mediated immune responses [33]. Studies such as these suggest that there are factors that may increase the risk of *M. bovis* infection in cattle; however, such studies have not evaluated the impact of such factors on shedding of *M. bovis*. Understanding the impact of such factors could be useful in decreasing transmission by isolating cattle in late parturition and avoiding the use of common maternity pens.

It has been stated that *M. bovis* bacteremia is not common in cattle and its occurrence may be associated with disseminated disease [34], although one study did not correlate disseminated disease with mycobacteremia [35]. Indeed, the isolation of culturable *M. bovis* from blood is rare in cases of natural or experimental infection [5,9,36,37]; however, using various molecular methods, *M. tuberculosis* complex DNA has been identified in variable numbers of naturally infected cattle, including those that are negative by conventional assays of cell-mediated immunity, including IGRA and TST [38,39,40,41,42].

Despite the lack of detectable *M. bovis* via PCR and culture methods in oronasal secretions and blood samples, the cell-mediated and humoral immune responses of the aerosol inoculated calves in this study are similar to those described in endobronchial infected calves during the first 10 weeks after infection [25]. This would suggest that the route of infection did not markedly influence the immune responses. In both studies, cell-mediated responses, as measured by IGRA were seen 2–3 weeks after infection and a small number of animals showed detectable humoral responses, as measured by IDEXX *M. bovis* ELISA 4–6 weeks after infection. Unlike cellular immune responses to *M. bovis*, humoral responses are believed to be greatest late in the course of disease and, therefore, negative antibody responses early in the disease are not surprising [43]. These data would suggest that *M. bovis* was found in sufficient numbers to elicit both cellular and humoral responses. Additionally, the presence of lesions consistent with tuberculous disease and bacterial burden in the mediastinal lymph nodes at necropsy provide further evidence of a successful infection model.

The current study utilizes a low-dose aerosol model of *M. bovis* infection to detect shedding of mycobacteria within the first 8 weeks of infection. This challenge model may more closely resemble field conditions of infection through aerosol transmission.

As reported above, this study is congruent with the inconsistent detection of *M. bovis* in naturally infected cattle and with experimental infection studies that use intrabronchial routes of infection. Altogether, these data demonstrate the challenges associated with the detection of *M. bovis* in bodily fluids, even when molecular assays with high sensitivity such as PCR are used. Further work is needed to develop assays to diagnose bTB through pathogen detection. If the future of bTB diagnosis is early pathogen detection, further work is needed to determine alternative samples for collection and/or alternative detection assays.

## Figures and Tables

**Table 1:** Real time PCR cycle threshold values (Ct) from nasal swabs collected at weekly intervals after experimental aerosol infection with *M. bovis*. Only animals with at least one positive result are listed. Animals not listed had no positive results.

Animal	Infection Status	Replicate	Weeks Post-Infection								
			0	1	2	3	4	5	6	7	8
68917	Infected	2	ND	ND	ND	ND	ND	ND	ND	ND	36.26
68211	Infected	2	ND	ND	ND	ND	ND	36.19	ND	ND	ND
69261	Infected	1	ND	ND	ND	ND	ND	ND	36.15	ND	ND
68779	Infected	3	ND	ND	ND	ND	ND	ND	36.25	ND	ND

ND = not detected, Ct value undetermined.

**Table 2:** Real time PCR cycle threshold values (Ct) from saliva samples collected at weekly intervals after experimental aerosol infection with *M. bovis*. Only animals with at least one positive result are listed. Animals not listed had no positive results.

Animal	Infection Status	Replicate	Weeks Post-Infection								
			0	1	2	3	4	5	6	7	8
68392	Infected	3	ND	37.49	ND	ND	ND	ND	ND	ND	ND
68299	Infected	1	ND	ND	ND	ND	ND	37.14	ND	ND	ND
66429	Infected	1	ND	ND	ND	ND	ND	ND	ND	34.67	ND
68917	Infected	2	ND	ND	ND	ND	ND	ND	ND	ND	36.26

ND = not detected, Ct value undetermined.

## Data Availability

The original contributions presented in this study are included in the article/Supplementary Material; further inquiries can be directed to the corresponding author/s.

## Supplementary Materials

The following supporting information can be downloaded at: https://www.mdpi.com/article/10.3390/vetsci11080357/s1, Figure S1: Interferon gamma release assay results of non-infected control cattle, Figure S2: IDEXX ELISA results of non-infected control cattle, Figure S3: Weights (gm) of caudal mediastinal lymph nodes from *M. bovis*-infected cattle and non-infected control cattle, Figure S4: Correlation matrices for each replicate evaluating correlation coefficients between lung lesion scores, caudal mediastinal lymph node weights, and bacterial burden (CFU/gm of *M. bovis*) in caudal mediastinal lymph nodes from *M. bovis*-infected cattle.
