# Supplementary material for: Pathogen Detection in Early Phases of Experimental Bovine Tuberculosis
Source: Vet Sci. 2024 Aug 7;11(8):357. doi: 10.3390/vetsci11080357 (PMC11359862; doi:10.3390/vetsci11080357)
Supplement: Supplementary file 1 [file vetsci-11-00357-s001.zip › vetsci-3080044-supplementary.pdf]

Pathogen Detection in Early Phases of Experimental Bovine Tuberculosis

Mitchell V. Palmer, Carly Kanipe, Soyoun Hwang, Tyler C. Thacker, Kimberly A. Lehman, Nicholas A. Ledesma, Kristophor K. Gustafson and Paola M. Boggiatto

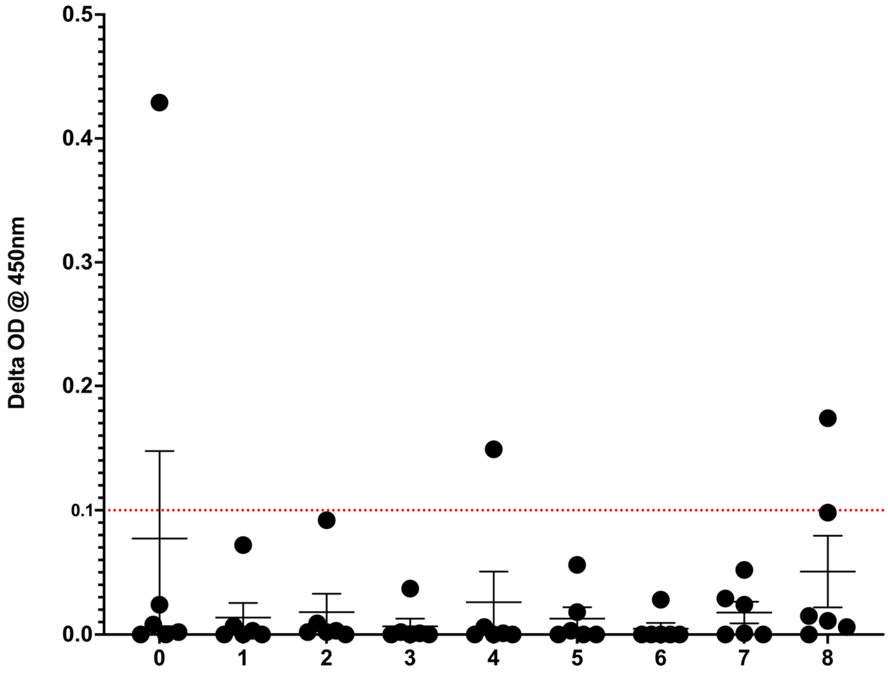

Figure S1. Interferon gamma release assay (IGRA) results from 6 non-infected control sampled at the same timepoints as cattle experimentally infected with aerosolized *M. bovis* prior to infection (week 0) and weekly thereafter for 8 weeks. Results are presented as mean (PPD-B −PPD-A; ΔOD) @ 450nm ± SEM. Cut-off for positive results is ΔOD ≥ 0.1 (dotted line).

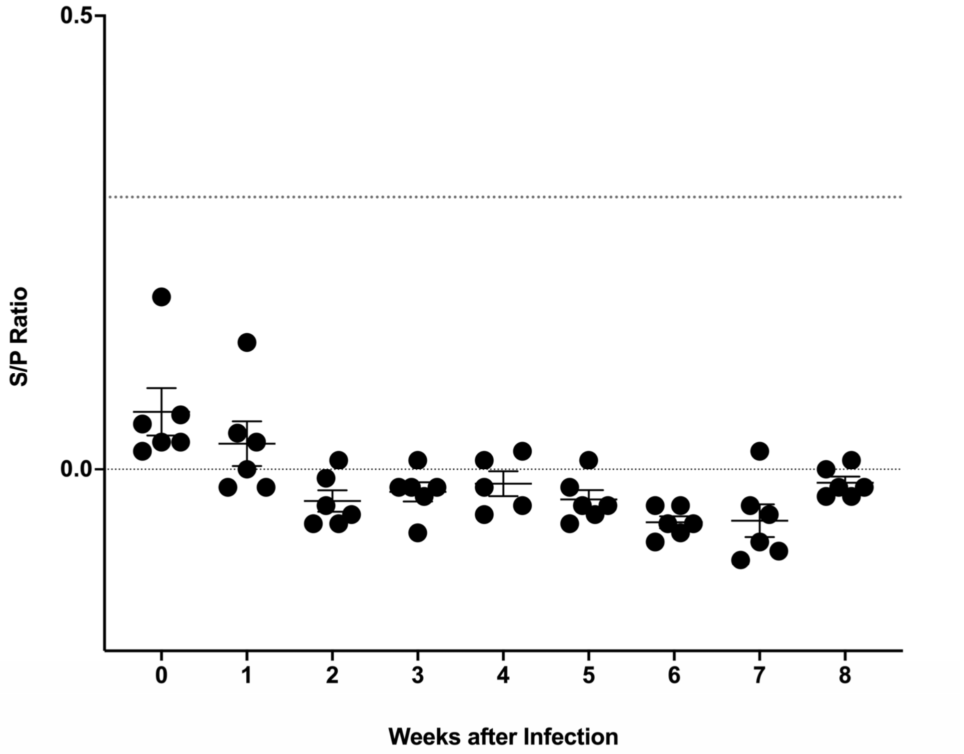

Figure S2. IDEXX ELISA results from 6 non-infected control cattle sampled at the same timepoints as cattle experimentally infected with aerosolized *M. bovis* (week 0) and weekly thereafter for 8 weeks. Results are presented as an S/P ratio ± SEM. A sample was considered positive if the S/P ratio was ≥ 0.30 (dotted line).

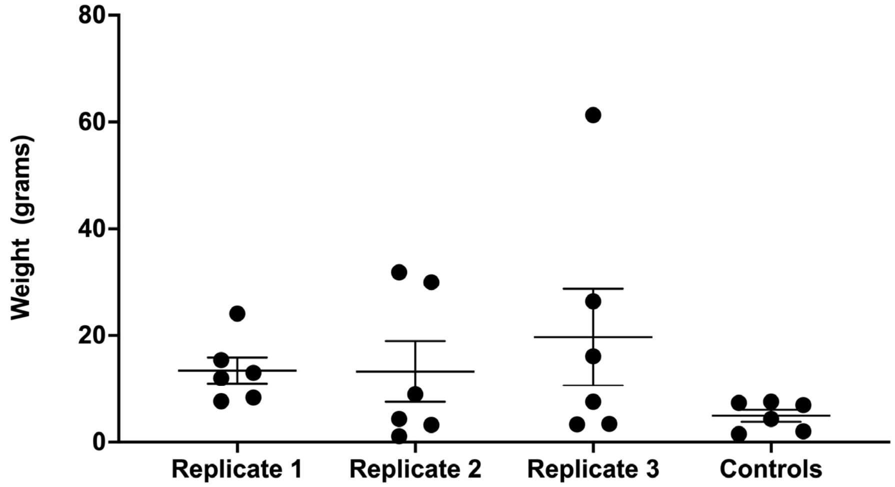

Figure S3. Weight (gm) of caudal mediastinal lymph nodes of 3 replicates of 6 cattle each experimentally inoculated with aerosolized *M. bovis* and 6 non-infected control cattle. Replicate 1 was euthanized and examined 268 to 281days after infection. Replicate 2 was euthanized and examined 329 to 336 days after infection, while replicate 3 was euthanized and examined 250 to 252 days after infection. Results are expressed as mean gm ± SEM.

A

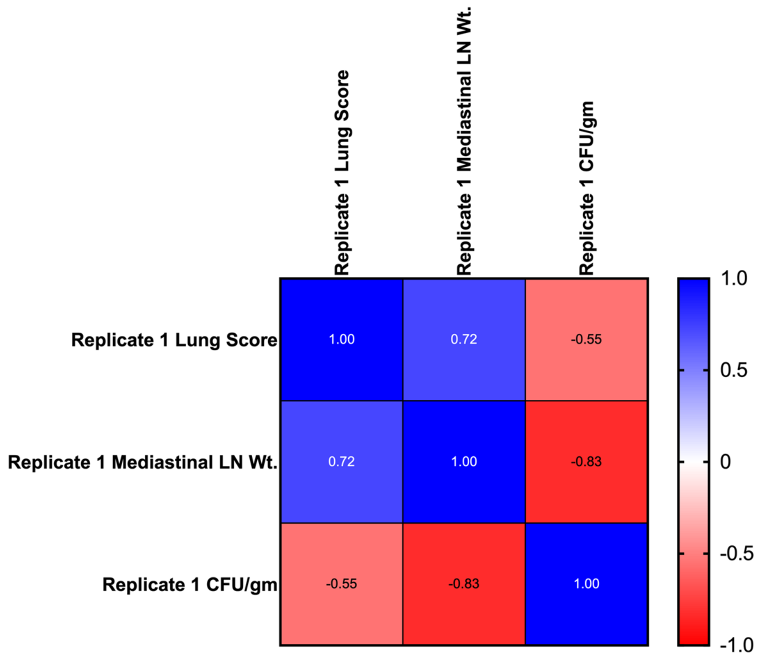
Replicate 1 Lung Score
Replicate 1 Mediastinal LN Wt.
Replicate 1 CFU/gm
Replicate 1 Lung Score
Replicate 1 Mediastinal LN Wt.
Replicate 1 CFU/gm
1.00
0.72
-0.55
0.72
1.00
-0.83
-0.55
-0.83
1.00
1.0
0.5
0
-0.5
-1.0

B

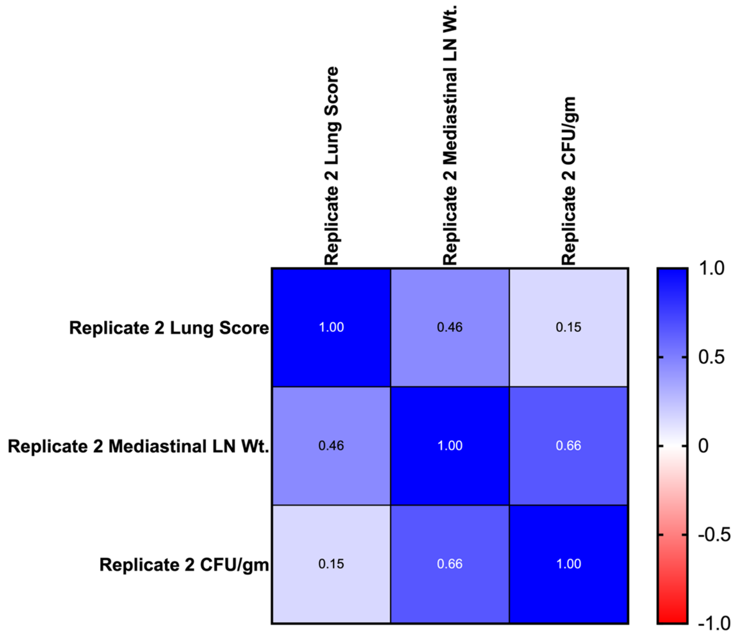
Replicate 2 Lung Score
Replicate 2 Mediastinal LN Wt.
Replicate 2 CFU/gm
Replicate 2 Lung Score
Replicate 2 Mediastinal LN Wt.
Replicate 2 CFU/gm
1.00
0.46
0.15
0.46
1.00
0.66
0.15
0.66
1.00
1.0
0.5
0
-0.5
-1.0

C

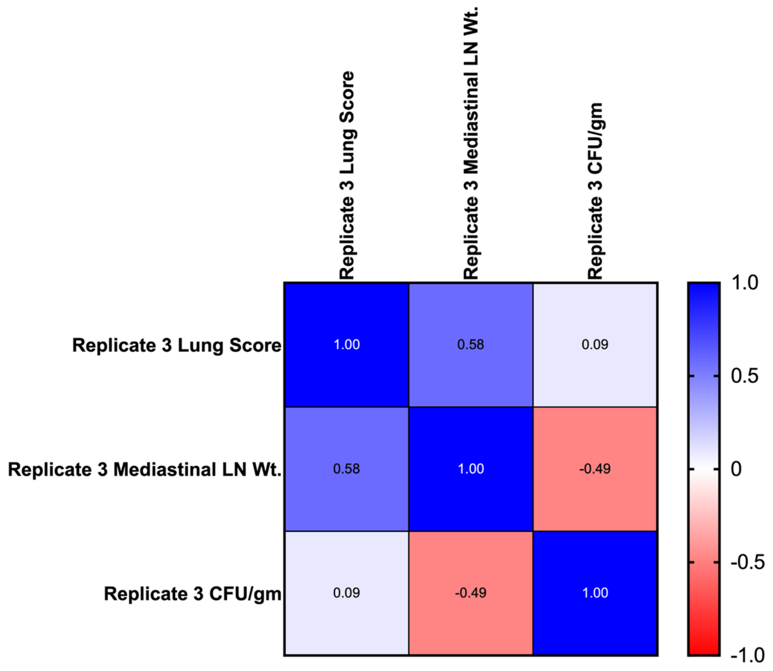

Figure S4. Spearman correlation matrix of lung lesion scores, caudal mediastinal lymph node weights (gm) and bacterial burden (CFU of *M. bovis* /gm) in caudal mediastinal lymph nodes of 3 replicates of 6 cattle each experimentally inoculated with aerosolized *M. bovis*. Replicate 1 was euthanized and examined 268 to 281days after infection. Replicate 2 was euthanized and examined 329 to 336 days after infection, while replicate 3 was euthanized and examined 250 to 252 days after infection. Values represent correlation coefficients (r) between variables.
